# The Efficacy of Ultrasound-Guided Thoracic Erector Spinae Plane Block for Perioperative Analgesia During Laparoscopic Bariatric Surgery in Super-Obese Patients: A Randomized Controlled Trial

**DOI:** 10.5812/aapm-170030

**Published:** 2026-06-30

**Authors:** Amany A Eissa, Mostafa Essam Abdelrahman Abdelmohsen, Marwa Zayed, Nashwa Nabil Mohamed Talaat, Mai Ahmed Ali, Yasmin Hassabelnaby, Fatma Elzahraa Mohamed Haggag, Mona Hamed Elsherbiny

**Affiliations:** 1 Department of Anesthesia, Surgical Intensive Care and Pain Medicine, Faculty of Medicine, Cairo University Cairo Egypt; 2 Department of Anesthesia Technology, College of Applied Medical Sciences in Jubail, Imam Abdulrahman Bin Faisal University Dammam Saudi Arabia

**Keywords:** Laparoscopic Bariatric Surgery, Thoracic Erector Spinae Plane Block, Analgesia, Super-obese Patients

## Abstract

**Background:**

Super-obese patients undergoing laparoscopic bariatric surgery are at an increased risk of opioid-related respiratory compromise, underscoring the need for opioid-sparing analgesia.

**Objectives:**

To compare bilateral ultrasound-guided thoracic erector spinae plane block (ESPB) and trocar-site local anesthetic infiltration with respect to perioperative opioid consumption and early postoperative pain.

**Methods:**

In this single-center randomized controlled trial, 60 adults aged 18 - 60 years with American Society of Anesthesiologists physical status III and a Body Mass Index (BMI) > 50 kg/m^2^ who were scheduled for laparoscopic sleeve gastrectomy or Roux-en-Y gastric bypass were randomized 1:1 to receive bilateral T7 ESPB with 0.25% bupivacaine (30 mL/side) or trocar-site infiltration with 0.5% bupivacaine (5 mL/site). The primary outcome was cumulative perioperative opioid consumption during the first 24 hours, expressed as an intravenous morphine-equivalent dose. Secondary outcomes included intraoperative fentanyl use, Visual Analog Scale pain scores at rest and during coughing, time to first rescue analgesia, time to ambulation, hemodynamics, the 15-item Quality of Recovery (15-item Quality of Recovery questionnaire (QoR-15)) score at 24 hours, and complications.

**Results:**

ESPB reduced intraoperative fentanyl requirements (56.7% vs 100%; total dose, 50 [25 - 75] vs 100 [100 - 212.5] µg; both P < 0.001) and cumulative 24-hour morphine-equivalent consumption (11 [9.75 - 13] vs 24.5 [22 - 33], mg; P < 0.001). Visual analog scale scores were lower with ESPB at rest at 4 and 8 hours and during coughing within the first 8 hours (all P < 0.001). Erector spinae plane block prolonged the time to first rescue analgesia (8 [8 - 12] vs 4 [4 - 8], h) and enabled earlier ambulation (4.07 ± 0.78 vs 4.60 ± 0.67 h; both P < 0.001). The QoR-15 score was higher with ESPB (118.8 ± 5.35 vs 106.6 ± 3.91; P < 0.001).

**Conclusions:**

In super-obese patients, thoracic ESPB provided a clinically meaningful opioid-sparing effect, improved early pain control and quality of recovery, and facilitated earlier mobilization following laparoscopic bariatric surgery.

## 1. Background

Rising global obesity is placing increasing strain on public health systems (1). Bariatric surgery remains an effective treatment, and procedure volumes continue to increase (2). Perioperative analgesia in patients with obesity is challenging because opioid-centered strategies may increase the risk of respiratory depression and pulmonary complications, underscoring the need for opioid-sparing techniques (3).

In ultrasound-guided erector spinae plane block (ESPB), local anesthetic is injected deep to the erector spinae muscle and spreads craniocaudally, providing somatic and visceral abdominal analgesia (3). Erector spinae plane block is simple and quick to perform (4) and has been used in thoracic, cardiac, breast, and abdominal surgery (5). However, its feasibility and effectiveness in patients with obesity remain uncertain.

In patients with obesity, peripheral nerve blocks are technically challenging because of difficult positioning and indistinct landmarks. Although ultrasound improves needle guidance and target identification, deeper targets and thicker tissue degrade image quality because of beam attenuation and artifacts, including speckle and clutter (5).

Only a few studies have evaluated ESPB in bariatric surgery (5-7), reporting opioid-sparing effects and improved pain control. However, to our knowledge, its efficacy has not been specifically examined in super-obese patients.

## 2. Objectives

To evaluate the effects of bilateral ultrasound-guided thoracic ESPB versus trocar-site bupivacaine infiltration on intraoperative and postoperative opioid requirements and pain control during the first 24 hours after bariatric surgery in super-obese patients.

## 3. Methods

### 3.1. Study Design and Setting

This single-center, parallel-group, assessor-blinded randomized controlled trial enrolled 60 adults of both sexes aged 18 - 60 years with a Body Mass Index (BMI) > 50 kg/m^2^ and American Society of Anesthesiologists physical status III (8) who were scheduled for elective laparoscopic sleeve gastrectomy or Roux-en-Y gastric bypass.

Preparatory work began at Cairo University Hospitals in March 2024, and the study was completed in June 2025. The Research Ethics Committee approved the study on February 20, 2024 (MD-15-2024), and the trial was prospectively registered on ClinicalTrials.gov on April 17, 2024 (NCT06377605). The study is reported in accordance with the Consolidated Standards of Reporting Trials (CONSORT) guidelines. All patients provided written informed consent.

### 3.2. Eligibility Criteria

Exclusion criteria were refusal of regional anesthesia; neurological or psychiatric disorders or poor cooperation; a high risk of obstructive sleep apnea (STOP-BANG score ≥ 3); previous bariatric surgery; concomitant laparoscopic procedures (e.g., cholecystectomy or hernia repair); anatomical abnormalities or skin lesions at the injection site; coagulopathy (international normalized ratio > 1.4 and/or platelet count < 100,000/µL); hepatic disease; malignancy; hypersensitivity to amide local anesthetics; and conversion to open surgery.

### 3.3. Randomization and Allocation

An independent investigator who was not involved in recruitment or data collection generated a computer-based block randomization sequence (block size, 4), allocating patients 1:1 to two groups of 30.

Allocation was concealed using sequentially numbered, opaque, sealed, tamper-proof envelopes prepared by an independent investigator and opened by the attending anesthesiologist immediately before the intervention.

Owing to the nature of the interventions, the anesthesiologist performing the block and managing anesthesia and the surgeon were not blinded. No sham procedure was performed; therefore, patients were not blinded. Postoperative pain, opioid consumption, and 15-item Quality of Recovery (15-item Quality of Recovery questionnaire (QoR-15)) scores were recorded by trained postanesthesia care unit (PACU) and ward staff who were unaware of group allocation. Identical, standardized anesthetic, analgesic, and rescue protocols were applied to both groups.

### 3.4. Intervention

#### 3.4.1. Erector Spinae Plane Block Group

All blocks were performed preoperatively according to a standardized protocol by a single anesthesiologist with more than 5 years of experience in regional anesthesia and more than 50 prior ESPB procedures. Correct placement required visible linear hydrodissection and local anesthetic spread beneath the erector spinae muscle. Cold sensation was tested bilaterally 20 minutes after injection, before induction. Block success was prospectively defined as bilateral loss of cold sensation covering at least the T5–T10 dermatomes (the surgical and trocar-site field) within 20 minutes; otherwise, the block was considered to have failed.

After receiving intravenous (IV) midazolam 1 mg, patients were placed in the lateral or seated position. A curvilinear ultrasound transducer (3 - 5 MHz) was placed sagittally at the T7 transverse process, approximately 3 cm lateral to the spinous process. After skin infiltration with 5 mL of 1% lidocaine, a 20-gauge blunt-tip needle (Pajunk Sonoplex) was advanced in-plane. Once hydrodissection with 5 mL of saline confirmed the target plane, 30 mL of 0.25% bupivacaine was injected under sterile conditions between the erector spinae muscle and the transverse process, and the procedure was repeated contralaterally (total dose, 150 mg). This fixed volume ensures adequate craniocaudal spread, consistent with published abdominal and bariatric ESPB protocols. Dosing by actual body weight was avoided because it would cause overdose and systemic toxicity in super-obese patients; the fixed 150 mg dose remained below the commonly cited adult maximum of 175 mg for plain bupivacaine (9, 10).

Predefined block-related complications (vascular puncture or intravascular injection, injection outside the intended plane, pneumothorax, hematoma, and injection-site infection) and signs of local anesthetic systemic toxicity (LAST) were monitored and documented.

#### 3.4.2. Infiltration Group

Patients in the infiltration group received 5 mL of 0.5% bupivacaine at each of five trocar sites before skin incision (total volume, 25 mL; total dose, 125 mg). Volumes differed because ESPB relies on high-volume interfascial spread, whereas infiltration targets individual trocar sites (11).

Both groups were prospectively monitored for LAST, including cardiovascular signs (bradycardia, conduction disturbances, and arrhythmias), using continuous electrocardiography (ECG), blood pressure monitoring, and pulse oximetry, and neurological signs (metallic taste, perioral numbness, tinnitus, dizziness, visual disturbance, agitation, tremor, and seizures). Plasma bupivacaine concentrations were not measured.

### 3.5. Anesthesia Technique

All patients were assessed in the preanesthesia holding area, including a review of their medical history, laboratory investigations, and fasting status, and the assigned intervention was explained.

After 18-gauge IV cannulation, standard monitoring (ECG, pulse oximetry, and noninvasive blood pressure) was applied, and baseline values were recorded. Intravenous ondansetron 8 mg was administered as premedication, and the ESPB group received bilateral ESPB before induction.

Anesthesia was induced with fentanyl 1 - 2 µg/kg (maximum 200 µg), propofol 2 mg/kg, and atracurium 0.5 mg/kg and maintained with isoflurane 1.5% in 50:50 oxygen–air plus atracurium 0.1 mg/kg every 30 minutes, with ventilation adjusted for normocapnia. The infiltration group received trocar-site infiltration, and all patients received IV paracetamol 1 g. An increase in heart rate (HR) or mean arterial pressure (MAP) > 20% above the pre-induction baseline was treated with fentanyl 50 µg IV. Ketorolac 0.5 mg/kg in 100 mL saline was administered 20 minutes before the end of surgery. Neuromuscular blockade was reversed with atropine 0.02 mg/kg and neostigmine 0.05 mg/kg, and patients were extubated and observed in the PACU for 60 minutes. Hypotension (MAP decrease > 20% or systolic pressure < 100 mm Hg) was treated with ephedrine 5 mg IV every 3 minutes as needed, and bradycardia (HR < 50 beats/min) was treated with atropine 0.5 mg IV.

### 3.6. Postoperative Care

In the PACU, pain was assessed using the Visual Analog Scale (VAS) 30 minutes after extubation; a VAS score > 3 was treated with IV nalbuphine 0.05 mg/kg, repeated after 30 minutes if pain persisted. On the ward, patients received scheduled IV paracetamol 1 g and IV ketorolac 30 mg every 8 hours.

Breakthrough pain (VAS score > 3) was managed identically in both groups with IV ketorolac 30 mg (maximum, 120 mg/day), followed, if pain persisted after 30 minutes, by IV nalbuphine 0.05 mg/kg (maximum, 6 mg per dose and 30 mg per 24 hours). Protocol adherence was documented for all cases.

### 3.7. Rationale for Nalbuphine Rescue Analgesia

As a κ-opioid agonist and μ-opioid antagonist, nalbuphine provides effective analgesia, including visceral analgesia, with a ceiling effect on respiratory depression and fewer μ-opioid-related adverse effects than pure μ-agonists. These properties are advantageous in super-obese patients at increased risk of postoperative hypoventilation (12, 13).

### 3.8. Data Collection and Measurement Tools

Demographic characteristics, outcome variables, and adverse events were recorded. Hemodynamics were recorded at baseline, 5 minutes after intubation, every 10 minutes intraoperatively, after extubation, and every 10 minutes for 30 minutes in the PACU. Visual analog scale scores were assessed at 30 and 60 minutes and at 2, 4, 8, 12, and 24 hours postoperatively (mild, 1 - 3; moderate, 4 - 6; severe, 7 - 10).

Total 24-hour opioid consumption (intraoperative fentanyl plus postoperative nalbuphine) was converted to IV morphine equivalents using ratios prespecified in the protocol from established equivalence references: fentanyl 100 µg = morphine 10 mg, and nalbuphine 10 mg ≈ morphine 10 mg.

### 3.9. Outcomes

The prespecified primary outcome was cumulative 24-hour perioperative opioid requirement (IV morphine equivalents). Secondary outcomes were intraoperative fentanyl requirement, VAS scores at rest and on coughing, time to first rescue analgesia, time to ambulation, perioperative hemodynamics, block-related complications, and total QoR-15 score at 24 hours. QoR-15 domain scores were exploratory, unadjusted for multiple comparisons, and interpreted as hypothesis-generating (5-7).

### 3.10. Sample Size

Sample size estimation was guided by Elgendy et al. (3), who reported a 24-hour morphine requirement of 24 ± 9 mg in a similar population. Fifty-four patients provided 80% power to detect a 30% reduction (two-sided α = 0.05); allowing for 10% attrition, 60 patients were recruited (30 per group). Calculations were performed using MedCalc® version 14.10.2.

### 3.11. Statistical Analysis

Analyses were performed using SPSS version 27 (IBM, Armonk, New York, United States). Normality was assessed using the Shapiro–Wilk test and visual inspection. Quantitative data are presented as mean ± standard deviation (SD) or median (interquartile range [IQR]) and were compared using the independent t-test or Mann–Whitney U test, respectively. Categorical data are presented as numbers (%) and were compared using the chi-square or Fisher’s exact test. Within-group repeated measurements were compared using repeated-measures analysis of variance (ANOVA) or the Friedman test, and the group-by-time interaction was assessed using mixed-design repeated-measures ANOVA. All tests were two-sided, with P < 0.05 considered significant.

## 4. Results

Of the 78 patients assessed for eligibility, 11 were excluded (6 did not meet the eligibility criteria, 3 had a high risk of obstructive sleep apnea on the STOP-BANG questionnaire, and 2 had previous bariatric surgery), and 7 declined participation. The remaining 60 patients were randomized equally to the ESPB and infiltration groups. No patients were lost to follow-up or excluded after randomization, and all were analyzed (Figure 1).

**Figure 1.**
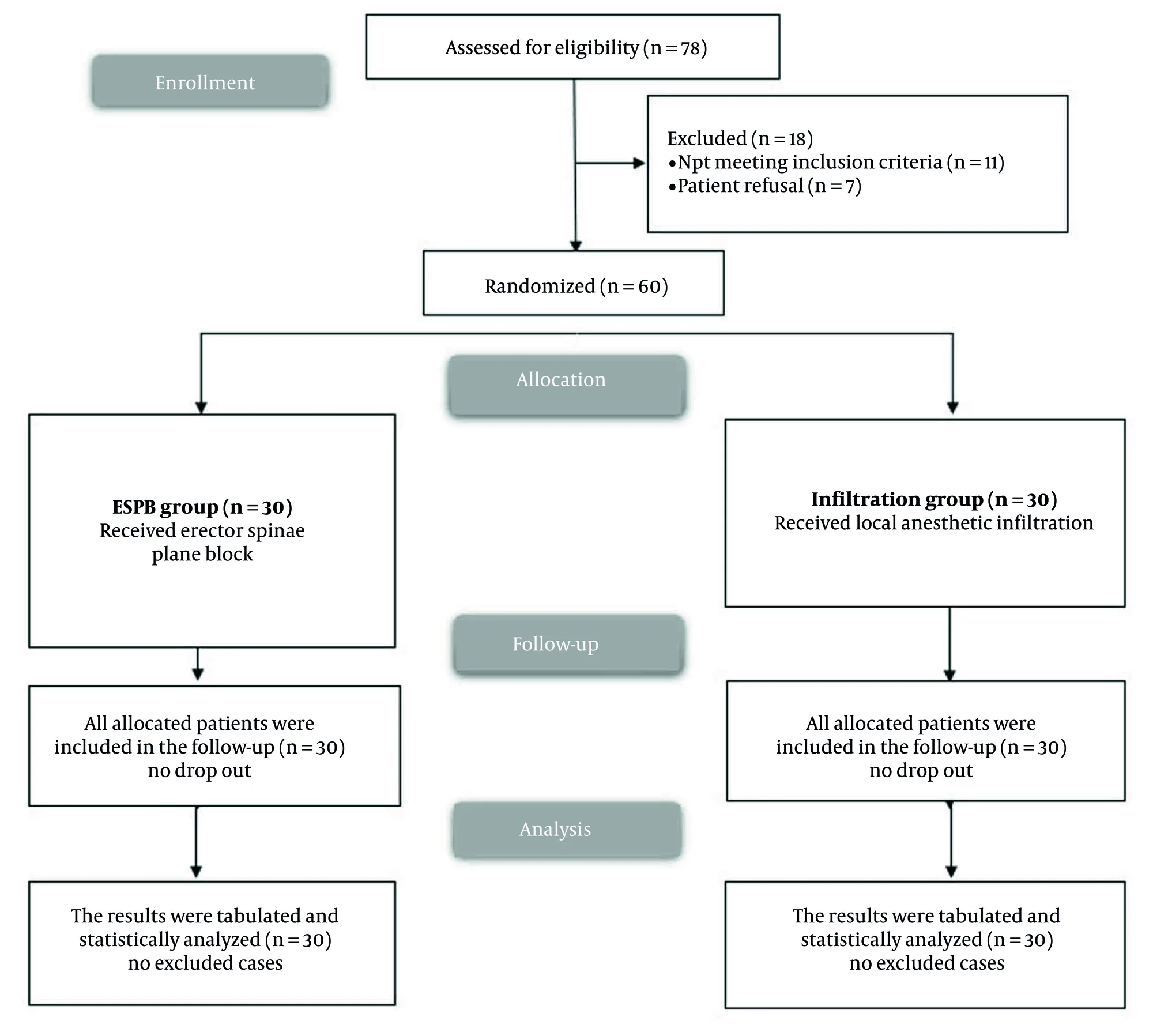
CONSORT flow diagram of patient enrollment, allocation, follow-up, and analysis.

Baseline characteristics were comparable between the ESPB and infiltration groups, including age (32.3 ± 8.14 vs 32.07 ± 7.24 years; P = 0.907), male sex (43.3% vs 36.7%; P = 0.598), and BMI (55.47 ± 2.85 vs 55.53 ± 2.89 kg/m^2^; P = 0.929). Sleeve gastrectomy was the most common procedure (86.7% vs 80%), with gastric bypass performed in 13.3% vs 20% of patients (P = 0.488).

Baseline MAP and HR were comparable between groups. Intraoperatively, both variables changed significantly over time in the ESPB group (P2 = 0.016 and 0.012, respectively) and the infiltration group (both P2 < 0.001). Compared with baseline, MAP decreased significantly at 5, 10, 20, 100, 120, and 130 minutes in the ESPB group but increased at 5 - 40 and 80 - 130 minutes in the infiltration group. HR decreased significantly only at 120 minutes in the ESPB group but was higher than baseline at 5 - 50 and 70 - 130 minutes in the infiltration group. The between-group interaction was significant for intraoperative MAP and HR (both P-int < 0.001). After extubation, neither variable changed significantly in the ESPB group (P2 = 0.72 and 0.161) or the infiltration group (P2 = 0.639 and 0.62), and the interaction was not significant (MAP, P-int = 0.444; HR, P-int = 0.145; Figure 2A and B).

**Figure 2.**
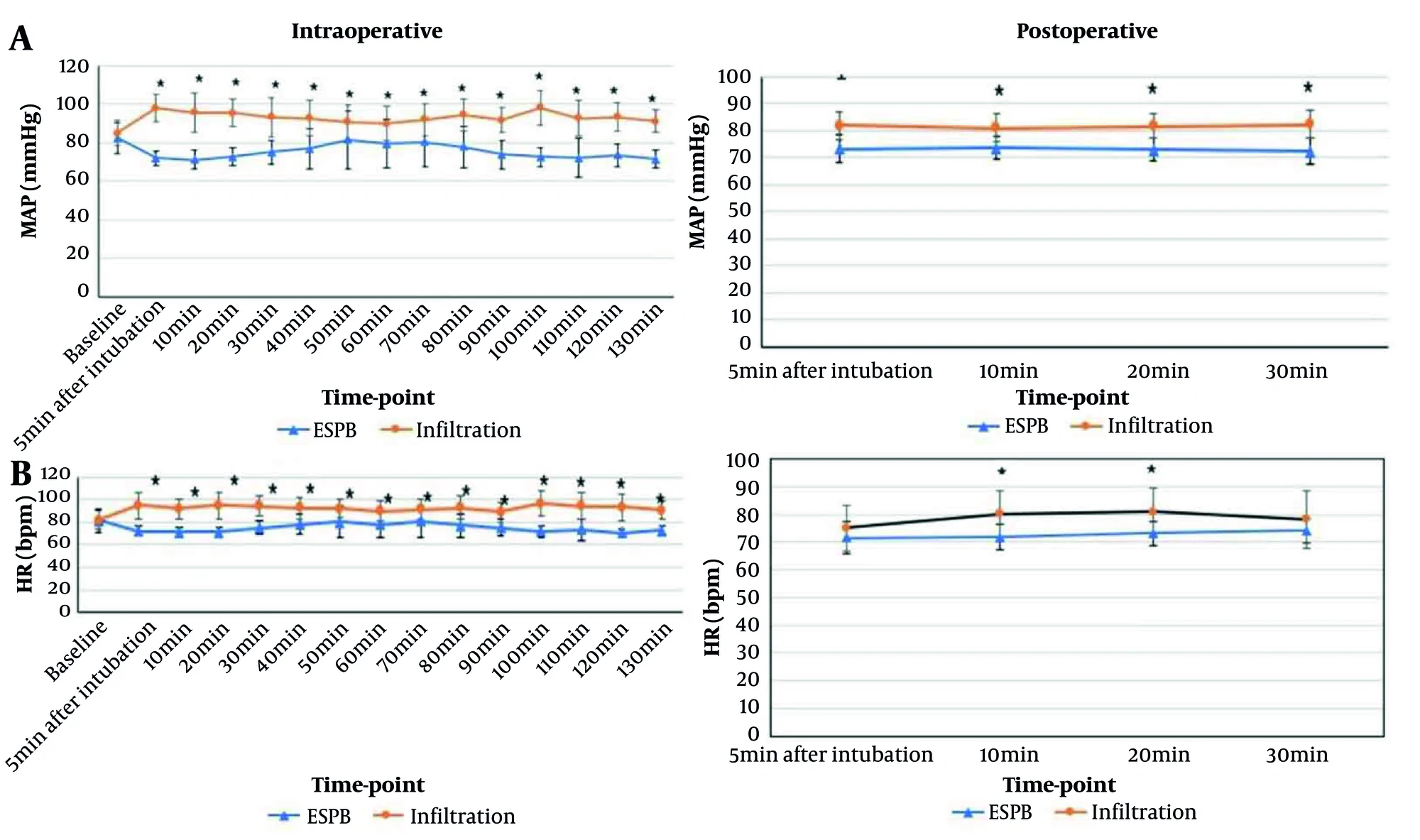
Perioperative hemodynamic changes in the studied groups: (A) mean arterial pressure and (B) heart rate (HR) at predefined intraoperative and post-extubation timepoints.

Postoperative VAS scores at rest and during coughing changed significantly over time in both groups (all P2 < 0.001). Compared with the earliest assessment, VAS scores at rest increased significantly from 2 hours onward in both groups, whereas VAS scores during coughing changed significantly from 4 hours onward in the ESPB group and from 2 hours onward in the infiltration group. The between-group interaction was significant for both outcomes (P-int < 0.001 for each; Figure 3, panels A-B).

**Figure 3.**
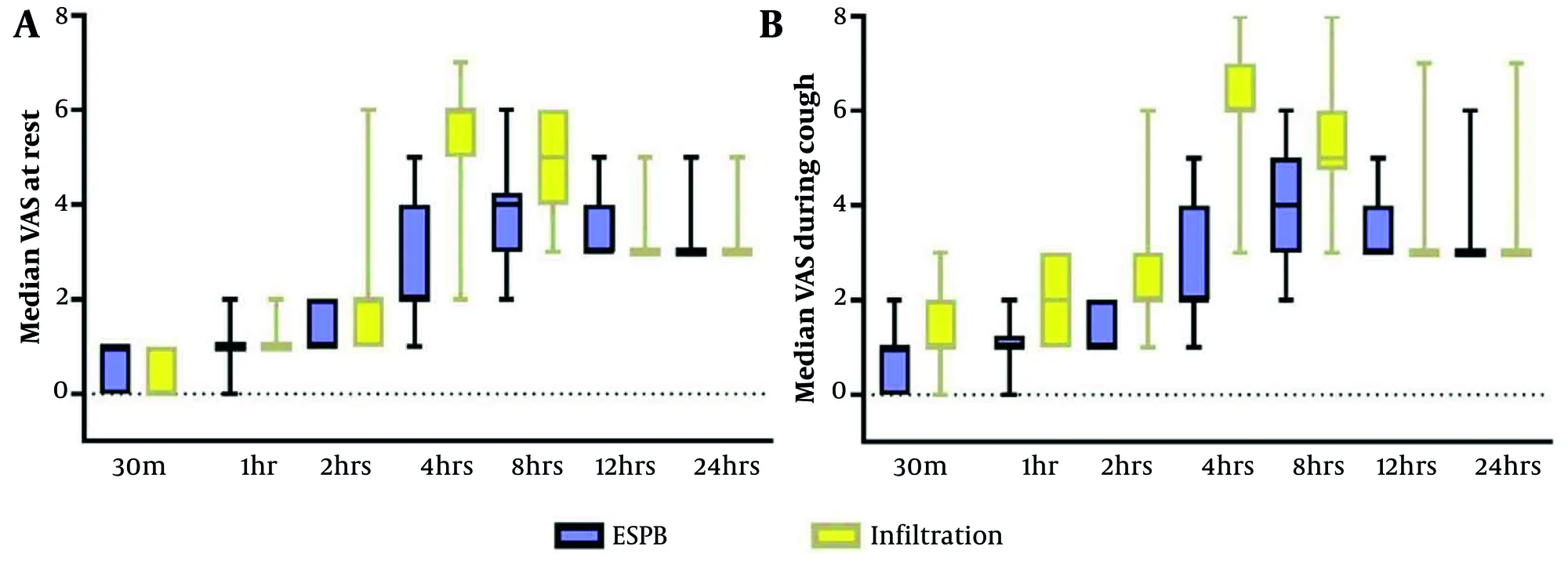
Postoperative Visual Analog Scale pain scores during the first 24 hours: (A) at rest and (B) during coughing.

All 30 ESPB patients achieved bilateral sensory blockade covering at least the T5–T10 dermatomes (100% success), with no failed or partial blocks and no repeat attempts.

Fewer ESPB patients required intraoperative fentanyl (56.7% vs 100%; P < 0.001), and the total intraoperative dose was significantly lower (P < 0.001). Median cumulative 24-hour morphine-equivalent consumption was also lower in the ESPB group (11 [9.75 - 13] vs 24.5 [22 - 33] mg; P < 0.001), representing a median reduction of 13.5 mg (approximately 55%). Patients in the ESPB group ambulated earlier and had a longer time to first rescue analgesia (both P < 0.001; Table 1).

**Table 1.** Perioperative Opioid Consumption and Early Recovery Outcomes in the Studied Groups ^a^

Outcomes	ESPB (n = 30)	Infiltration (n = 30)	P-Value ^b^
**Patients requiring intraoperative fentanyl**	17 (56.7%)	30 (100%)	< 0.001
**Total intraoperative fentanyl dose (µg)**	50 (25 - 75)	100 (100 - 212.5)	< 0.001
**Cumulative 24-hour morphine-equivalent dose (mg)**	11 (9.75 - 13)	24.5 (22 - 33)	< 0.001
**Time needed to start walking (h)**	4.07 ± 0.78	4.60 ± 0.67	< 0.001
**Time for first rescue analgesia (h)**	8 (8 - 12)	4 (4 - 8)	< 0.001

^a^ Values are expressed as mean ± SD, median (IQR), or n (%), as appropriate. Abbreviations: ESPB, erector spinae plane block; IQR, interquartile range; SD, standard deviation.

^b^ Significant P-value.

The total QoR-15 score was significantly higher in the ESPB group (118.8 ± 5.35 vs 106.6 ± 3.91; P < 0.001). Exploratory domain analyses also favored the ESPB group, with higher Part A scores for breathing comfortably, feeling rested, sleeping well, independent personal hygiene, communicating with family or friends, staff support, resuming usual activities, and feeling comfortable and in control, and less severe pain, nausea/vomiting, worry/anxiety, and sadness/depression in Part B (all P < 0.05; Table 2).

**Table 2.** Quality of Recovery Questionnaire Results of the Studied Groups ^a^

Item	ESPB (n = 30)	Infiltration (n = 30)	P-Value
**Part A**			
How have you been feeling in the last 24 hours?			
Able to breathe easily	8.1 ± 0.71	7.6 ± 0.56	0.004^b^
Able to enjoy food	5.57 ± 1.3	5.8 ± 1.32	0.494
Feeling rested	8.17 ± 1.05	6.8 ± 0.96	< 0.001^b^
Have had a good sleep	7.83 ± 0.95	7 ± 0.59	< 0.001^b^
Able to look after personal toilet and hygiene unaided	7.67 ± 0.61	6.8 ± 0.71	< 0.001^b^
Able to communicate with family or friends	8.4 ± 0.56	7.6 ± 0.5	< 0.001^b^
Getting support from hospital, doctors and nurses	8.43 ± 0.5	8.17 ± 0.46	0.037^b^
Able to return to work or usual home activities	7.67 ± 0.55	6.6 ± 0.56	< 0.001^b^
Feeling comfortable and in control	7.9 ± 0.84	6.7 ± 0.75	< 0.001^b^
Having a feeling of general well-being	7.97 ± 0.85	7.7 ± 0.95	0.257
**Part B**			
Have you had any of the following in the last 24 hours?			
Moderate pain	8.13 ± 0.9	7.63 ± 0.85	0.031^b^
Severe pain	9.17 ± 0.7	7.23 ± 1.1	< 0.001^b^
Nausea or vomiting	7.7 ± 0.75	6.93 ± 0.78	< 0.001^b^
Feeling worried or anxious	7.93 ± 0.78	7.13 ± 0.9	0.001^b^
Feeling sad or depressed	8.13 ± 0.68	6.9 ± 0.92	< 0.001^b^
Total score	118.8 ± 5.35	106.6 ± 3.91	< 0.001^b^

^a^ Values are expressed as mean ± SD. In Part A, each item is scored from 0 (none of the time; poor) to 10 (all of the time; excellent). In Part B, each item is reverse-scored from 10 (none of the time; excellent) to 0 (all of the time; poor). Higher total QoR-15 scores indicate better recovery. Abbreviations: ESPB, erector spinae plane block; QoR-15, 15-item Quality of Recovery questionnaire; SD, standard deviation.

^b^ Significant P-value.

No patient developed signs or symptoms of LAST, block-related complications (pneumothorax, vascular puncture, hematoma, or injection-site infection), or severe opioid-related respiratory adverse events during the 24-hour observation period.

## 5. Discussion

With the increasing global prevalence of obesity, bariatric surgery has become more common. Perioperative analgesia in patients with obesity is challenging because opioids increase respiratory and pulmonary risks (14). Opioid-sparing options after laparoscopic surgery include regional blocks and intraperitoneal local anesthetics; for example, Shahinfar et al. (15) evaluated intraperitoneal bupivacaine with a lung recruitment maneuver for postoperative shoulder pain.

Ultrasound-guided ESPB provides somatic and visceral analgesia and may reduce opioid requirements, but technical limitations in patients with obesity can affect block performance (16-18). Evidence in super-obese patients (BMI > 50 kg/m^2^) is limited; therefore, we compared bilateral ESPB with trocar-site local anesthetic infiltration in this population.

In this study, ESPB was superior to trocar-site infiltration in reducing cumulative perioperative opioid consumption and improving early analgesia and recovery. The median reduction of 13.5 mg (approximately 55%) in 24-hour morphine-equivalent consumption exceeded the clinician-perceived minimal clinically important difference of approximately 10 mg of IV morphine or a relative reduction of 20%–40% (19). However, this reflects superiority over the selected infiltration regimen rather than absolute efficacy versus placebo, sham block, or other regional techniques.

Erector spinae plane block was also associated with better patient-reported recovery, including a higher total QoR-15 score, generally favorable exploratory domain scores, delayed rescue analgesia, and earlier ambulation. These findings suggest that its opioid-sparing effect may translate into improved early recovery; however, the domain results remain exploratory.

These findings are consistent with those of bariatric trials in patients with obesity. Karaveli et al. (20) reported effective ESPB analgesia during laparoscopic sleeve gastrectomy. In patients with BMI ≥ 40 kg/m^2^, Mostafa et al. (6) found lower early VAS scores, lower intraoperative fentanyl and 24-hour morphine use, and a longer time to first opioid requirement with ESPB. Zengin et al. (21) similarly reported reduced intraoperative opioid use, better pain control, and greater hemodynamic stability in morbidly obese patients.

In other laparoscopic procedures, Khalil et al. (22) found lower pain scores, a longer time to first morphine administration, and lower morphine use with ESPB than with an oblique subcostal transversus abdominis plane block in cholecystectomy. Rahimzadeh et al. (23) compared ESPB with retrolaminar block after upper abdominal laparoscopic surgery.

Similarly, Fu et al. (24) reported less pain, lower analgesic requirements, and earlier mobilization after hepatectomy, and Singh et al. reported reduced 24-hour morphine consumption after modified radical mastectomy (25).

Visual analog scale scores during coughing were significantly lower with ESPB than with infiltration at 30 minutes and at 1, 2, 4, and 8 hours. Similarly, Şafak et al. (26) reported lower VAS scores during coughing at 2, 4, 6, and 12 hours with bilateral T12 ESPB than with control in 116 women undergoing elective cesarean delivery. In contrast, Chen et al. (27) reported higher VAS scores with ESPB than with paravertebral block, likely reflecting a more anatomically targeted comparator than wound infiltration.

### 5.1. Limitations

This study has limitations. First, the single-center design may limit generalizability. Second, the interventions were not volume- or dose-matched (ESPB: 150 mg in 60 mL; infiltration: 125 mg in 25 mL); although this reflected their different anatomical requirements, not all differences can be attributed solely to the regional technique. Third, the intraoperative anesthesiologist and surgeon were aware of allocation, and no sham procedure was performed; therefore, patients and intraoperative clinicians were not blinded, although outcome assessors were blinded and intraoperative fentanyl was protocol-driven. Fourth, excluding patients at high risk of obstructive sleep apnea limits applicability to the broader super-obese population. Fifth, follow-up was limited to 24 hours, precluding assessment of longer-term pain, late complications, or readmission. Sixth, ESPB is operator-dependent, and the success achieved by an experienced anesthesiologist may not be reproducible elsewhere. Finally, the study was powered only for the primary outcome, and QoR-15 domain analyses were unadjusted and exploratory.

### 5.2. Conclusions

In super-obese patients undergoing laparoscopic bariatric surgery, bilateral ultrasound-guided thoracic ESPB was superior to trocar-site bupivacaine infiltration in reducing cumulative perioperative opioid consumption and improving early pain control, with delayed rescue analgesia, earlier ambulation, a higher total QoR-15 score, and no LAST or major block-related complications within 24 hours. Multicenter trials including patients at high risk for obstructive sleep apnea and using matched comparator regimens are warranted.

## Data Availability

The dataset presented in the study is available on request from the corresponding author during submission or after publication.

## Clinical Trial Registration Code:

Clinical Trial Code: NCT06377605

(Link: https://clinicaltrials.gov/study/NCT06377605)
